# Diversity of Bradyrhizobia in Subsahara Africa: A Rich Resource

**DOI:** 10.3389/fmicb.2018.02194

**Published:** 2018-09-20

**Authors:** Jann Lasse Grönemeyer, Barbara Reinhold-Hurek

**Affiliations:** Department of Microbe-Plant Interactions, Faculty of Biology and Chemistry, Center for Biomolecular Interactions Bremen, University of Bremen, Bremen, Germany

**Keywords:** *Bradyrhizobium*, diversity, Subsahara Africa, Namibia, inoculant, temperature tolerance

## Abstract

Making use of biological nitrogen fixation (BNF) with pulses and green manure legumes can help to alleviate nitrogen deficiencies and increase soil fertility, problems faced particularly in smallholder agriculture in Subsahara Africa (SSA). The isolation of indigenous rhizobia provides a basis for the formulation of rhizobial inoculants. Moreover, their identification and characterization contribute to the general understanding of species distribution and ecology. Here we discuss global species discovery of *Bradyrhizobium* spp. Although recently the number of validly published *Bradyrhizobium* species is rapidly increasing, their diversity in SSA is not well-represented. We summarize the recent knowledge on species diversity in the *Bradyrhizobium yuanmingense* lineage to which most SSA isolates belong, and their biogeographic distribution and adaptations. Most indigenous rhizobia appear to differ from species found on other continents. We stress that an as yet hidden diversity may be a rich resource for inoculant development in future. As some species are exceptionally temperature tolerant, they may be potential biofertilizer candidates for global warming scenarios.

## Introduction

In the past 50 years, increases in crop yield have been striking in some regions, e.g., particularly rice yields in Asia, due to green revolution. However, this high-input approach has been less successful in Sub-Saharan Africa (SSA) with its hugely variable environmental, climatic and cultural conditions (Rudebjer et al., 2013). The predominant agricultural practice based on improved varieties of common staple crops in high-input systems has not well-succeeded to address food insecurity and malnutrition in Sub-Saharan Africa, where the prevalence of undernourishment is still at 20.8%, not having decreased since 2010 (FAO, 2017). As exemplified for many SSA areas, agriculture in the Okavango region is largely dominated by smallholder and subsistence farming. Recent surveys conducted at sites in Angola, Namibia and Botswana revealed that 99, 88, and 59% of the households, respectively, practice arable agriculture (Domptail et al., 2013; Große et al., 2013; Kowalski et al., 2013). Variability of yields, risk for crop failure, limited financial resources, and low-fertility N-poor soils are among the contributing factors to food insecurity in these rainfed agriculture systems (Pröpper et al., 2010). These risks are predicted to increase in SSA due to climate change. Projections of consequences of climate change at the local scale indicated that the Kavango basin will become warmer (1.5–2.5°C), and obtain less mean annual precipitation (50–100 mm) until 2045 (Pröpper et al., 2015).

Making use of biological nitrogen fixation (BNF) of root nodule symbioses with pulses and legume green manure can help to alleviate nitrogen deficiencies and increase soil fertility (Pule-Meulenberg et al., 2010). The application of rhizobial inoculant carrying highly effective rhizobia can boost BNF and is regarded as a cost-effective and sustainable approach to increase yields in N-limited agricultural systems with low productivity (Dakora and Keya, 1997; Mpepereki and Pompi, 2003). However, established inoculant strains often fail when transferred to regions featuring environmental conditions dissimilar to their original habitat, presumably due to poor persistence and competitiveness (Roughley, 1970; Mpepereki and Pompi, 2003; Zhang et al., 2003; Law et al., 2007). Many agriculturally important legumes enter a symbiotic association with rhizobia of the genus *Bradyrhizobium*. Especially pulses commonly used by smallholders in SSA, cowpea (*Vigna unguiculata*), Bambara groundnut (*Vigna subterranea*), and peanut (*Arachis hypogaea*), are nodulated by *Bradyrhizobium* spp. Here, we will first highlight that the diversity of bradyrhizobia – as potential adapted inoculants - is largely underexplored in SSA, and provide recent insights into their biogeography and diversity.

## Global species discovery of *Bradyrhizobium* spp.

**Figure 1:**
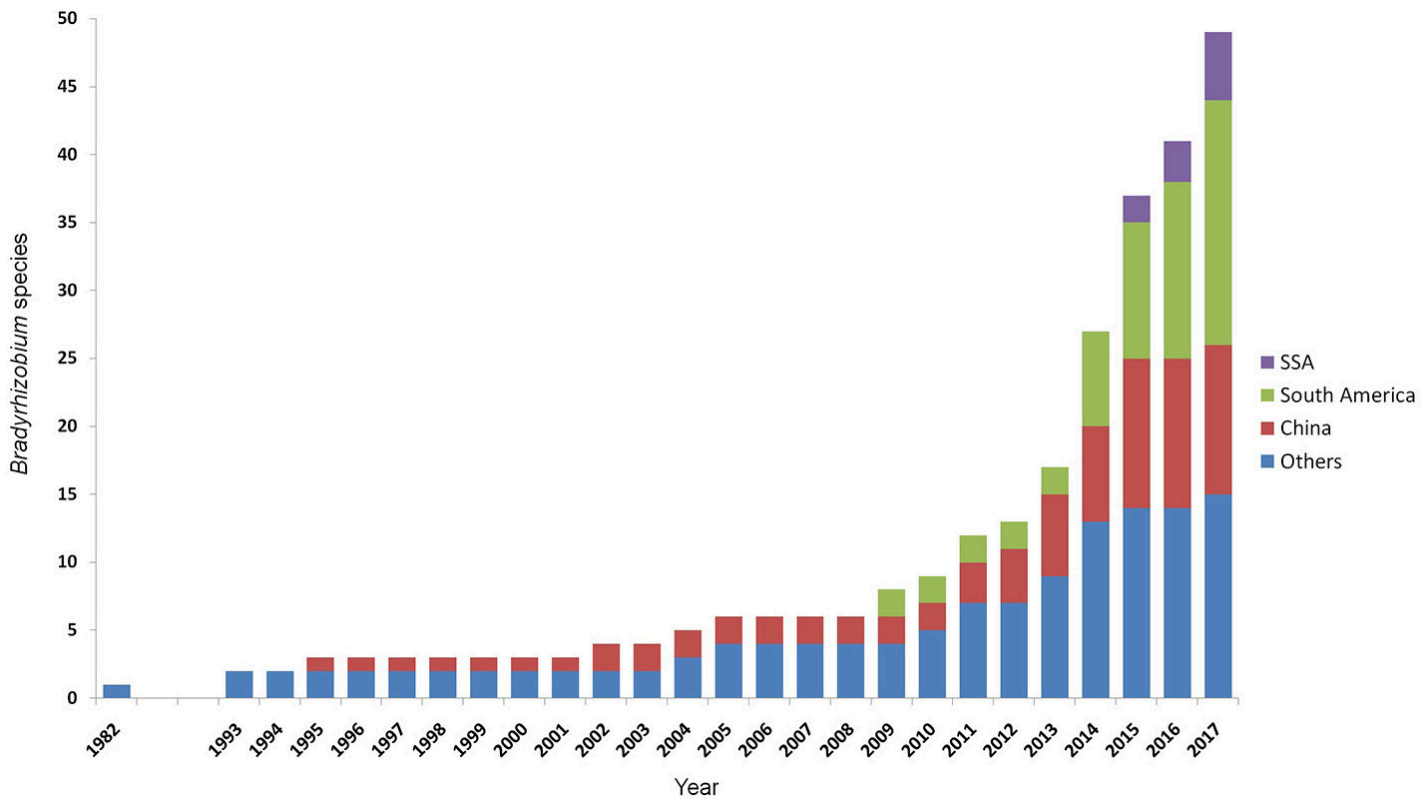
Increase of described species of the genus *Bradyrhizobium* with time. Data includes all species officially listed (Parté, 2014; de Lajudie and Young, 2017), and the effectively, but not validly, published species “*B. arachidis*,” “*B. valentinum*,” “*B. brasilense*,” “*B. sacchari*,” “*B. centrolobii*,” “*B. macuxiense*,” and “*B. shewense*” (Wang et al., 2013; Durán et al., 2014; Aserse et al., 2017; da Costa et al., 2017; de Matos et al., 2017; Michel et al., 2017).

For a long time the species diversity inside *Bradyrhizobium* remained underexplored due to the exceptional conservation of the 16S rRNA gene sequence that is routinely used as a marker for species discrimination (van Berkum and Fuhrmann, 2000). The high genospecies diversity inside *Bradyrhizobium* was first discovered in a DNA-DNA hybridization study by Willems et al. (2001). With the help of alternative markers and multilocus sequence analysis (MLSA) (Stepkowski et al., 2005; Vinuesa et al., 2005b), species delineation became more feasible. Now the number of validly published bradyrhizobial species is rapidly increasing since 2012 (Figure 1) and currently counting 42 validly published species, more than half of which have been published since 2014 (Parté, 2014; de Lajudie and Young, 2017). Approximately one third of the described species originates from South America, one third from other regions, and a large number from China, while only few originate from SSA (Figure 1). As yet, only five species from SSA have been published: *Bradyrhizobium kavangense* (Grönemeyer et al., 2015b), *Bradyrhizobium namibiense* (Grönemeyer et al., 2017), *Bradyrhizobium subterraneum* (Grönemeyer et al., 2015a), *Bradyrhizobium vignae* (Grönemeyer et al., 2016), and “*Bradyrhizobium shewense*” (Aserse et al., 2017). Thus, only few genotypes for development of effective inoculants for agricultural crops are taxonomically well-described and thereby well-comparable.

Within the sub-Saharan regions, the plant species richness and endemism is particularly high in the Cape Floristic Region, the East Coast near Mozambique, and the Congo-Zambezi watershed (Linder, 2001). Among *Leguminosae / Fabaceae*, roughly 1,500 were yet alone considered in Southern Africa (Trytsman et al., 2016). SSA being the center of origin for many legumes, these regions might entail a high diversity of effective microsymbionts (Pule-Meulenberg, 2014). The full potential of SSA indigenous legumes may not yet have been recognized, although they can be predicted as valuable germbank for possible agricultural use in arid and semi-arid regions (Sprent et al., 2010). Uncovering the full diversity and species richness of the respective symbionts may provide a vast resource for inoculant development for legume crops and forage plants. As especially in these regions, smallholder farming is widespread that could greatly profit from adapted inoculant technology, future research should focus on unraveling the putative biodiversity of rhizobia and particularly *Bradyrhizobium* in SSA.

## Putative *Bradyrhizobium* diversity to be uncovered in SSA

The increased number of bradyrhizobial species allowed several studies to uncover a geographic distribution. Most of our knowledge about rhizobia and their biogeography is based on studies conducted in Asia, Europe and the Americas (Pule-Meulenberg, 2014). Information on SSA rhizobia is limited despite SSA regions presumably entail a high microsymbiont diversity that is favored by at least three factors: First, SSA is characterized by heterogeneous soils and climates, providing diverse habitats (Petersen et al., 2010; Gröngröft et al., 2013; Wade et al., 2014). Second, rhizobial diversity may be higher in arid and semi-arid regions frequently found in SSA, as observed for Senegal (Wade et al., 2014) or Brazil (Martins et al., 1997). It has been suggested that the selection pressure on rhizobia may lead to the evolution of stress tolerant strains which could partly explain the increased diversity observed in water limited environments. Third, Africa is the center of origin of many legumes (including cowpea and Bambara groundnut) and a rich diversity of wild legume species exists (Pule-Meulenberg, 2014; Lemaire et al., 2015).

The tribe Crotalarieae, for instance, is largely endemic to SSA (14 endemic genera comprising over 1,000 species) and known for its high microsymbiont diversity, including *Bradyrhizobium, Rhizobium, Methylobacterium, Microvirga, Mesorhizobium, Ensifer*, and *Burkholderia* (Aserse et al., 2012; Sprent et al., 2013; Ndungu et al., 2018). Earlier studies using DNA fingerprinting already indicated a high microsymbiont diversity in SSA regions (Botha et al., 2004; Law et al., 2007). To date, only a small number of surveys used MLSA to uncover the microsymbiont diversity in SSA. The few studies focusing on “cowpea group” rhizobia (from agricultural plants) spanned the countries of Botswana and South Africa (Steenkamp et al., 2008), Botswana, South Africa, and Ghana (Pule-Meulenberg et al., 2010), Senegal (Wade et al., 2014), Namibia and Angola (Grönemeyer et al., 2014), Ghana and South Africa (Puozaa et al., 2017), Mozambique (Chidebe et al., 2018), Kenya (Ndungu et al., 2018), and Ethiopia (Degefu et al., 2018). Their main findings were in general agreement: First, almost all detected genotypes presumably represented yet unknown species (Steenkamp et al., 2008; Grönemeyer et al., 2014; Wade et al., 2014; Degefu et al., 2018). Second, genotype occurrence strongly relied on the geographic location (Steenkamp et al., 2008; Pule-Meulenberg et al., 2010; Grönemeyer et al., 2014; Wade et al., 2014). Third, the highly diverse genotypes were mainly assigned to a sub-generic group, the *Bradyrhizobium yuanmingense* lineage (Wade et al., 2014), forming a clade with *B. vignae* and *B. subterraneum*, but not to the sub-generic group of *Bradyrhizobium japonicum* (Steenkamp et al., 2008; Grönemeyer et al., 2014; Wade et al., 2014).

**Figure 2:**
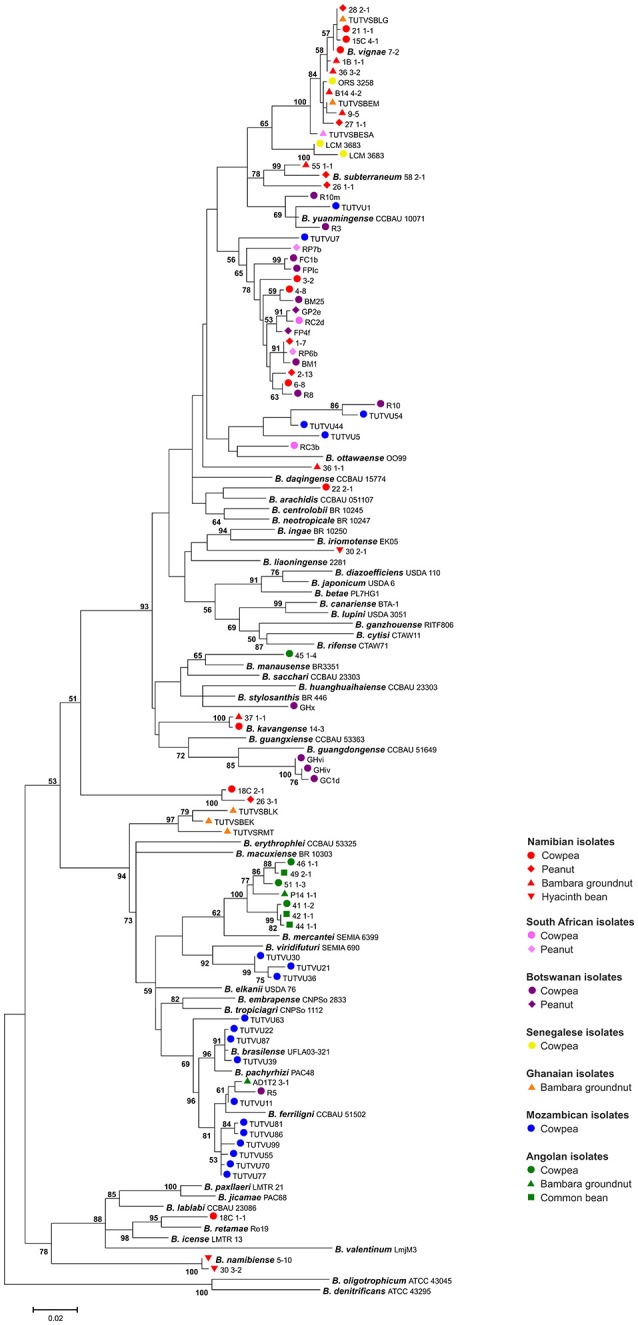
Maximum likelihood-based phylogeny inferred from *glnII-recA* sequences of SSA bradyrhizobia and species type strains. SSA isolates from agricultural plants (see legend) obtained in five African studies are represented (Steenkamp et al., 2008; Grönemeyer et al., 2014; Wade et al., 2014; Puozaa et al., 2017; Chidebe et al., 2018). Considerably shorter sequences were excluded, and representatives were selected for identical sequences. The tree was calculated from 751 positions using the General Time Reversible model. A bootstrap value is indicated when the associated taxa clustered together in ≥50% of 500 pseudoreplicates. The scale bar indicates the number of substitutions.

The expectation of high bradyrhizobial species diversity from agricultural plants in SSA is supported by the phylogeny of published isolates of the abovementioned studies (Figure 2). Matching the previous findings, many isolates form distinct phylogenetic clusters and could not be assigned to recognized species. In the rare cases where African genotypes cluster with named species, sequence divergence is high, indicating different genospecies affiliations. Thus most indigenous rhizobia appear to differ from species found on other continents.

The observed high rhizobial diversity entails the discovery of yet unknown species. Remarkably is, however, that recognized species are virtually absent in SSA. Apart from the geographic location, this observation might be related to the natural selectivity of the sampled legume hosts and the lack of taxonomic studies. Relatively few studies focused on natural microsymbionts of cowpea and especially Bambara groundnut (Pule-Meulenberg, 2014; Puozaa et al., 2017). The first validly published species from this host, *B. subterraneum* (Grönemeyer et al., 2015a) was mainly isolated from Bambara groundnut, and also from peanut. Though the number of studies is limited, Bambara groundnut is apparently a promiscuous host. In our cross-inoculation experiments, almost the whole spectrum of tested bradyrhizobia, including reference species, induced effective nodulation on Bambara groundnut (Grönemeyer et al., 2014). Only five out of 26 cowpea rhizobia failed to nodulate Bambara groundnut in a study in Zimbabwe (Mpepereki et al., 1996), albeit indicating a certain degree of selectivity. Most other detected phylotypes include isolates from cowpea. This is not surprising since cowpea is one of the most promiscuous legumes (Lewin et al., 1987; Bala and Giller, 2001). Cowpea rhizobia collections are usually highly diverse (Grönemeyer et al., 2014; Wade et al., 2014), and several studies even reported strains from genera other than *Bradyrhizobium* to nodulate cowpea (Mpepereki et al., 1996; Martins et al., 1997). Hence, cowpea isolates obtained at a specific site largely reflect the local abundance of competitive bradyrhizobial microsymbionts, providing a solid basis to study bradyrhizobial biodiversity. Several studies surveyed the rhizobial diversity associated with cowpea in China (Zhang et al., 2008), India (Appunu et al., 2009), Japan (Sarr et al., 2011), Mexico (Ormeno-Orrillo et al., 2012), and Spain (Bejarano et al., 2014). In contrast to African studies, phylotypes could be clearly assigned to named species. Almost all cowpea isolates from Japan represented either *B. japonicum, Bradyrhizobium diazoefficiens, B. yuanmingense*, or *Bradyrhizobium elkanii*, whereas *B. yuanmingense* dominated in India and *Bradyrhizobium cytisi* and *Bradyrhizobium canariense* in Spain, for instance.

## Linkage of genotype occurrence and geographic location

The increased number of bradyrhizobial species allowed several studies to uncover a geographic distribution, and biogeography could be linked to different variables such as climate (Vinuesa et al., 2008; Risal et al., 2010; Adhikari et al., 2012), soil pH (Li et al., 2011; Adhikari et al., 2012), water regime (Wade et al., 2014), salinity and soil potassium content (Zhang et al., 2011; Chen et al., 2016), and geographic isolation (Stepkowski et al., 2012). Biogeography is apparently related to adaptations at multiple levels, ranging from climate to micro niche (Wade et al., 2014), and a biogeographic distribution of SSA isolates from agricultural plants is also reflected in Figure 2. A survey on the impact of climate, for instance, indicated that *B. japonicum, B. canariense*, and *B. yuanmingense* are mainly found in temperate regions in the Northern Hemisphere, in Mediterranean regions, or in the subtropics and tropics, respectively (Vinuesa et al., 2008). Consistently, *B. japonicum* was shown to be less competitive in soybean nodulation under higher temperatures (Suzuki et al., 2014). The relevance of adaptation at a more local level is indicated by the prevalent detection of *Bradyrhizobium liaoningense* in alkaline soils (Li et al., 2011; Adhikari et al., 2012), contrasting both *Bradyrhizobium pachyrhizi* and *B. canariense* that are primarily found in acid soils (Vinuesa et al., 2005a; Grönemeyer et al., 2014). Species abundance might be further conditioned by physico-chemical parameters like soil osmotic strength, as indicated in a recent survey in Senegal (Wade et al., 2014).

Thus, the assignment of a bradyrhizobial strain to a distinct species can generate information on adaptational properties, which in turn account for a strain's suitability to serve as inoculant at a particular site. As species delineation is mainly based on similarity of conserved genes differences, adaptational properties may be further refined by accessory genes. To date, however, information on bradyrhizobial species biogeography in SSA regions is limited. Species may be restricted to certain geographic locations, but others may have spread among multiple regions and continents, with soil-contaminated seeds or artificial inoculation facilitating long-distance dispersal (Perez-Raminez et al., 1998). Host control and preferential selection can provide competitive advantages and thus constitute key drivers for the successful invasion into new geographic regions, especially when rhizobia and their hosts were co-introduced (Heath and Tiffin, 2009; Porter et al., 2011; Hollowell et al., 2016).

The uniqueness of SSA rhizobia might rather be attributed to their exclusion or the enrichment of other species by more selective and better studied legumes of temperate regions. However, many SSA rhizobia were found to possess the genomic background to produce highly decorated Nod factors, presumably allowing a broad host-range (Steenkamp et al., 2008). Nod factors are lipochitooligosaccharides “decorated” by diverse modifications, that induce early responses of the symbiotic interation in the legume host. Symbiotic genes are readily exchanged between bradyrhizobial species (Horn et al., 2014; Hollowell et al., 2016). Moreover, most bradyrhizobial lineages feature a broad host range, without strong barriers to adapt their chromosomal backgrounds to novel hosts (Parker, 2015). Consequently, host plant selectivity may in general have a minor impact on species abundance (Hollowell et al., 2016), and it is more likely that the observed biogeography is due to an adaptation of species to soil-climatic factors. Consistently, many cowpea and Bambara groundnut isolates effectively nodulate peanut and hyacinth bean (Grönemeyer et al., 2014). Thus, the range of sampled legume hosts is obviously not a major determinant of the observed species uniqueness.

Alternatively, unique soil-climatic conditions in SSA might have played a major role, according to the Baas-Becking hypothesis “everything is everywhere, but the environment selects.” However, several studies in SSA explored the rhizobial diversity under highly variable soil-climatic conditions (Grönemeyer et al., 2014; Wade et al., 2014). Moreover, several regions in southern Africa and Australia exhibit very similar soils and climates, as illustrated by the issue that weeds are easily exchanged (Kottek et al., 2006; Sprent et al., 2013). Nevertheless, their native floras are very different (Sprent et al., 2013). Consequently, geographic isolation and adaptation of rhizobia to various soil-climatic conditions and undomesticated legume hosts might have favored the evolution of diverse endemic species. Geographic isolation of SSA rhizobia was indicated in a previous study (Steenkamp et al., 2008): The authors found that, despite being extensively exchanged via horizontal gene transfer, all bradyrhizobial *nodA* sequences detected in SSA cluster in one of the seven recognized major clades (Stepkowski et al., 2007), contrasting Asian and American (but not European) *nodA* sequences which distribute over various clades. Recently, 16 *nodA* clades have been described, where clade III with the majority of SSA isolates is cosmopolitan, members being widespread in sub-Saharan Africa, the Americas, Australia and in southern and eastern Asia (Aserse et al., 2012; Beukes et al., 2016). Since the clade II strains include North African and European ones the African *nodA* clade might be ancient and spread to other continents until the Sahara desert formed. The resulting geographic isolation then prevented the import of *nodA* lineages (or rhizobial species) that evolved on other continents (Stepkowski et al., 2007). Conversely, species which evolved in SSA did not spread to other continents and were thus not detected in the many surveys conducted outside Africa.

## Temperature tolerance of bradyrhizobia in global climate change scenarios?

Geographic distribution might also be explained by adaptation at higher levels such as regional climatic conditions (Vinuesa et al., 2008; Wade et al., 2014). An initial survey using multilocus sequence analysis to assess bradyrhizobial biogeography found that *B. japonicum, B. diazoefficiens* (former *B. japonicum* Ia, Delamuta et al., 2013), and *B. elkanii* are very widespread across the Northern Hemisphere and are thus detected in more temperate regions (Vinuesa et al., 2008). Most studies conducted in SSA (see also Figure 2) pointed out that indigenous rhizobia were not assigned to the clade of *B. japonicum*. *B. japonicum* presumably evolved in regions outside SSA. Here, it adapted to more temperate and wet regions, thus probably lacking the prerequisite of higher level adaptation to many African climates. Moreover, a survey on soybean rhizobia along a climate gradient in Nepal revealed that roughly half of the isolates from temperate regions were related to *B. japonicum*, which was absent in subtropical regions (Risal et al., 2010). Indeed, the temperature tolerance for growth of *Bradyrhizobium* spp. varies greatly. Table 1 compares published maximum growth temperatures with geographic distribution, indicating that *B. japonicum* and closely related *B. diazoefficiens* and *Bradyrhizobium lupini* are all widespread in more temperate regions of both hemispheres, while phylotypes from warmer climates show a higher temperature tolerance. Many strains of *B. japonicum* grow best at 28°C (Munévar and Wollum, 1981). The genus *Bradyrhizobium* was indicated to optimally grow at 25–30°C, maximal 33–35°C (Kuykendall, 2005). In contrast, many SSA phylotypes still grow at 38°C, *B. vignae* exhibiting an exceptional high temperature tolerance growing above 40°C (Table 1; Grönemeyer et al., 2014, 2016). The known geographic range of *B. vignae* includes regions Namibia, Senegal, also Ghana and South Africa (Puozaa et al., 2017), Southern India and Northern Australia (Table 1 and Figure 2). A common feature of the regions may be climatic conditions. According to the Köppen-Geiger climate classification (Kottek et al., 2006), strikingly, climate of Namibia and Senegal is largely categorized as BSh (arid to semi-arid, steppe climate, hot), and Ghana, Southern India and Northern Australia are classified as Aw (tropical; hot with pronounced dry seasons). Thus, *B. vignae* is probably competitive in hot regions with seasonal drought, matching its exclusively high temperature tolerance. Furthermore, another phylotype cluster SA-3 (Steenkamp et al., 2008) represented by e.g., strain 1–7 from Namibia (Figure 2) intermingled with isolates obtained from Botswana and South Africa, as implying that this heterogeneous cluster is widespread in parts of southern Africa. Climate in Botswana is mostly given the same category as the Okavango region, and occurrence extends to regions of warm climate such as Senegal, Ethiopia, and Southern China (Table 1). Especially in regions with periodic harsh heat, temperature tolerance may be a decisive advantage in competition with other rhizobia, ensuring better persistence in soils.

**Table 1:** Maximum growth temperature (MGT) and geographic occurrence of selected *Bradyrhizobium* species and African phylotypes.

**Species/Phylotype**	**MGT^a^**	**Occurrence^b^**
*B. vignae*	40°C	Namibia (Kavango), Senegal, Ghana, Southern India, Northern Australia
*B. subterraneum*	38°C	Namibia (Kavango), Botswana (Notwane), South Africa (Taung), Northern Australia (Kununurra), Western Australia (Carnarvon), Peru
*B. kavangense*	38°C	Namibia (Kavango)
22 2-1	38°C	Namibia (Kavango), Northeastern Brazil (Bahia), Argentina (Cordoba)
45 1-4	38°C	Angola
*B. yuanmingense*	38°C	China (Hebei, Anhui, Sichuan, Hubei, Peking, Guangxi, Xinjiang, Henan, Laixi Country, Jiangsu), Taiwan, India (Thar desert, Madhya Pradesh, Tamil Nadu, Karnataka, Andhra Pradesh), Thailand (Uttradit, Lampang), Myanmar (Shan State), Vietnam, Southern Japan (Okinawa) Northern Australia (Kununurra), Bostwana (Rasesa), Northern Peru, Northern Ghana, South Africa (Taung), Senegal, and more
18C 2-1/26 3-1	38°C	Namibia (Kavango)
*B. namibiense*	37°C	Namibia (Kavango)
*B. ganzhouense*^c^	37°C	Southern China (Ganzhou)
36 1-1^c^	35°C	Namibia (Kavango)
SA-3 (3-2/1-7)	35°C	South Africa (Roodeplaat, Taung), Botswana (Maun, Rasesa, Francistown, Gaborone), Namibia (Kavango), Ethiopia, Senegal, Southern China
*B. diazoefficiens*	< 37°C	USA (North Carolina, Maryland, Mississippi, Iowa), Canada (Quebec, Ontario), Japan (Kyushu, Yamagata, Fukushima, Kumamoto, Hokkaido, Kagoshima, Miyagi, Niigata), China (Heilongjiang, Chengdu, Hubei, Anhui, Guangdong, Guangxi), Nepal (Kathmandu, Khumaltar, Khadichaur), Brazil, and more
*B. japonicum*	< 37°C	Japan, China (Heilongjiang, Chengdu, Guangdong, Guangxi, Sichuan, Anhui), Nepal (Kathmandu), USA (Mississippi, Maryland), Canada (Ontario, Quebec), Argentina, Brazil, South Africa (Mpumlanga)
*B. lupini*^c^	< 37°C	USA (Georgia, California), Spain (Canary Islands, Llombai), Northern Tunisia, Southern Australia (Esperance, Carrabin), England (Rothamsted Research)
30 2-1	< 35°C	Namibia (Kavango), Southern India (Karnataka)
51 1-3/42 1-1	< 35°C	Angola, Malaysia (Luasong), Southeastern Brazil (Seropedica), Mexico (Veracruz)
GHx^c^	Unknown	South Africa, Mexico (Veracruz)
GHiv^c^	Unknown	South Africa
TUTVSBEK^c^	Unknown	Ghana, Mozambique, South Africa, Nigeria, Ethiopia, Southern China, Myanmar, Taiwan, India, USA, and more
TUTVU36^c^	Unknown	Mozambique, Venezuela, Brazil, Mexico
AD1T2 3-1	Unknown	Angola, South Africa, Ethiopia, Ivory Coast, Brazil, Argentina, Mexico, China, Malaysia
TUTVU77^c^	Unknown	Mozambique, South Africa, Ethiopia, Brazil (Porto Trombetas), Mexico, Costa Rica, Malaysia (Luasong), South Korea (Cheongju), China, Myanmar, USA (North Carolina), Canada (Quebec)

a *Data from Grönemeyer et al. (2014) and Delamuta et al. (2013)*.

b *Based on ITS and glnII sequence identities of ≥ 98% in Genbank*.

c *No ITS sequence data available*.

## Prospects

It became apparent that the vast diversity of *Bradyrhizobium* species in SSA is as yet underestimated. As regional strains may be developed into adapted inoculants for pulses and green manure, research in diversity, and characterization of nodule symbionts in SSA should be intensified. Particularly the high temperature tolerance of some African *Bradyrhizobium* species makes them potential candidates for application in global climate change scenarios that predict temperature increases. Future research should also address the molecular basis for the unusual temperature tolerance.

## Author contributions

Both authors wrote the manuscript. JG reviewed the literature, gathered the information about species distribution and prepared the figures. BR-H organized the manuscript content.

### Conflict of interest statement

The authors declare that the research was conducted in the absence of any commercial or financial relationships that could be construed as a potential conflict of interest.
